# A Heterogeneous Viscosity Flow Model for Liquid Transport through Nanopores Considering Pore Size and Wettability

**DOI:** 10.3390/molecules29133176

**Published:** 2024-07-03

**Authors:** Yilin Chang, Yapu Zhang, Zhongkun Niu, Xinliang Chen, Meng Du, Zhengming Yang

**Affiliations:** 1 University of Chinese Academy of Sciences, Beijing 100049, China; changyilin21@mails.ucas.ac.cn (Y.C.); niuzhongkun19@mails.ucas.ac.cn (Z.N.); chenxinliang20@mails.ucas.ac.cn (X.C.); 2 Institute of Porous Flow and Fluid Mechanics, Chinese Academy of Sciences, Langfang 065007, China; zhangyapu69@petrochina.com.cn; 3 Sinopec North China Petroleum Bureau, Zhengzhou 450006, China; 4 Research Institute of Petroleum Exploration & Development, PetroChina Company Limited, Beijing 100083, China

**Keywords:** confined effect, flow suppression, boundary region, wettability, viscosity

## Abstract

The confinement effect in micro- and nanopores gives rise to distinct flow characteristics in fluids. Clarifying the fluid migration pattern in confined space is crucial for understanding and explaining the abnormal flow phenomena in unconventional reservoirs. In this study, flow characteristics of water and oil in alumina nanochannels were investigated with diameters ranging from 21 nm to 120 nm, and a heterogeneous viscosity flow model considering boundary fluid was proposed. Compared with the prediction of the HP equation, both types of fluids exhibit significant flow suppression in nanochannels. As the channel size decreases, the deviation degree increases. The fluid viscosity of the boundary region displays an upward trend as the channel size decreases and the influence of the interaction between the liquid and solid walls intensifies. The thickness of the boundary region gradually decreases with increasing pressure and eventually reaches a stable value, which is primarily determined by the strength of the interaction between the liquid and solid surfaces. Both the pore size and wettability are essential factors that affect the fluid flow. When the space scale is extremely small, the impact of wettability becomes more pronounced. Finally, the application of the heterogeneous flow model for permeability evaluation has yielded favorable fitting results. The model is of great significance for studying the fluid flow behavior in unconventional reservoirs.

## 1. Introduction

With the continuous growth of global oil and gas demand and the constant decline in conventional oil and gas production, unconventional oil and gas with great resource potential is gradually entering a period of active development [1,2]. Unconventional oil and gas resources mainly include shale oil, shale gas, tight oil, tight gas, etc. Unconventional reservoirs typically have poor physical properties, with porosity less than 10% and overburden permeability less than 0.1 mD [3]. They contain a large number of nanoscale pores and throats, with the main pore size ranging from 40 to 700 nm in tight sandstone and from 5 to 200 nm in shale [4,5]. Clarifying the fluid flow laws in nanochannels can provide an explanation for the anomalous flow phenomena in unconventional reservoirs and serve as a basis for determining parameters in related mathematical models for unconventional reservoirs [6,7,8].

Based on current experimental research on flow phenomena in nanochannels, both flow enhancement and flow suppression phenomena are possible when compared with theoretical equations such as the Hagen–Poiseuille equation and the Lucas–Washburn equation [9,10,11]. Holt, Majumder, and Whitby et al. found that the experimental flow rates of carbon nanotubes are significantly higher than those predicted by traditional models, with a difference of 4–5 orders of magnitude [12,13,14]. Secchi et al. pointed out that the slip length in carbon nanotubes can reach up to 300 nm with a channel size of 15 nm [15]. Meanwhile, Kelly et al. observed that the migration distance of isopropanol in nanochannels is at least five times smaller than predicted by the Lucas–Washburn equation [16]. Tomo et al., using transmission electron microscopy, discovered a very thin but highly stable water film on the hydrophilic carbon nanotube walls, with a thickness of approximately 1–7 nm [17]. Influenced by factors such as channel material, surface wettability, roughness, fluid type, and environmental temperature, fluid flow in nanochannels exhibits various flow characteristics [18,19,20,21].

When the spatial scale decreases to the nanoscale, the interaction between the fluid and the solid wall has a significant influence on the flow [22]. The fluid molecules present an ordered stacking state near the wall, with a layered structure of about 3–4 layers [23,24]. Due to the presence of this stacked structure, there are variations in the density and viscosity of the fluid near the wall compared to the bulk phase [25]. Consequently, the nanochannel can be divided into two regions: the bulk region and the boundary region. As the viscosity of the fluid decreases in the boundary region, rapid transport phenomena occur within the nanochannels [26,27]. Wu et al. suggested that the viscosity of the boundary region is influenced by the wettability of the wall. When the wall transitions from hydrophilic to hydrophobic, the viscosity of the fluid in the boundary region gradually decreases [28,29]. Despite ongoing research efforts, significant debate persists regarding the mass transfer characteristics of fluids in nanoconfined spaces. Thus, the development of thermodynamic and dynamic theoretical models tailored to nanoconfined fluids remains a critical challenge in the field of nanofluidics.

Research on fluid flow phenomena in nanochannels is typically fundamental, and experiments are conducted under simplified conditions to minimize environmental interference [30]. The flow rate of fluid in a single nanochannel deviates from conventional theoretical predictions, exhibiting significant nonlinear seepage characteristics in porous media and requiring a starting pressure gradient [31,32,33]. The key to applying basic research to the interpretation of flow phenomena in rocks lies in translating the migration laws obtained from experimental or simulation methods into relevant mathematical models that describe fluid migration in confined spaces [34,35].

In this paper, firstly, the flow behaviors of oil and water phases in nanochannels of various scales were investigated and the experimental flow rates with the theoretical flow rates predicted by the Hagen–Poiseuille equation were compared. A heterogeneous flow model was established considering the boundary fluid and viscosity and thickness variations of the fluid within the boundary region were analyzed individually. On the basis of confined channel research, an application example of a heterogeneous viscosity model in the study of seepage laws in tight reservoirs was presented. This research contributes to enhancing our understanding of fluid migration patterns in confined spaces and provides explanations for abnormal flow phenomena in unconventional reservoirs.

## 2. Results

### 2.1. Relationship between Experimental and Theoretical Flow Rate

**Figure 1:**
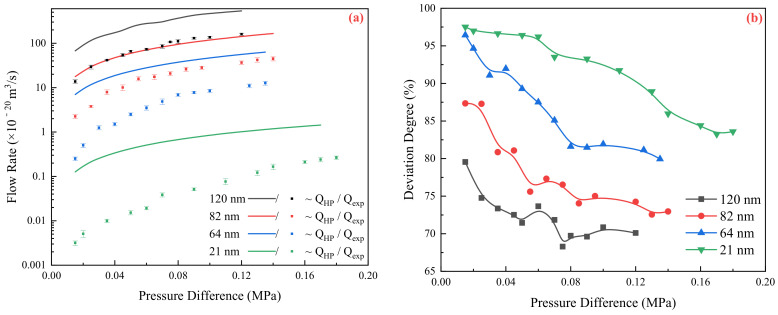
Flow characteristics of deionized water in nanochannels: (**a**) Comparison of theoretical and experimental flow rates under different pressures; (**b**) Deviation degree of flow in channels of different scales.

**Figure 2:**
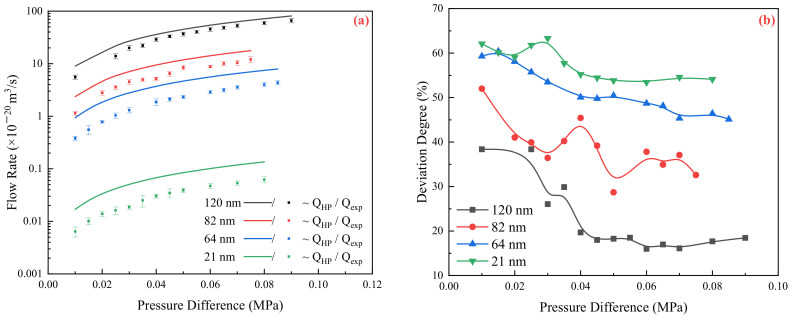
Flow characteristics of silicon oil in nanochannels: (**a**) Comparison of theoretical and experimental flow rates under different pressures; (**b**) Deviation degree of flow in channels of different scales.

The relationship between pressure and flow rate was determined for different pressure differences, pore sizes, and flow media, as shown in Figure 1 and Figure 2.

The data presented in Figure 1a illustrate the comparison between the theoretical flow rate and the experimental flow rate at different pressures. It is evident that as the channel size reduces to the nanoscale, the experimental flow deviates from the theoretical flow rate. The degree of deviation tends to increase with smaller channel sizes. Moreover, it can be observed that at low pressures, the flow exhibits nonlinear characteristics. As the pressure increases, the flow rate gradually becomes more linear and the deviation degree decreases. Eventually, the flow reaches a relatively stable state. The flow of water in nanochannels exhibits significant inhibition. The flow deviation degree is 70.1% when the channel size is 120 nm. As the channel size decreases to 21 nm, the deviation degree reaches 83.6%. These findings highlight the impact of channel size and pressure on flow behavior, particularly in nanoscale systems.

As illustrated in Figure 2, both oil and water demonstrate similar flow phenomena in confined spaces, which also restricts the movement of oil. As the pressure increases, the flow deviation degree gradually decreases and then stabilizes. On the other hand, as the channel size decreases, the flow deviation degree increases. It is noted that when the flow becomes stable and the pressure and flow rate maintain a linear relationship, the deviation degree of the oil phase is significantly smaller than that of the water phase. For example, when the channel scale is 120 nm, the final deviation degree of the oil phase is 18.5%, which is much less compared to the 70.1% deviation observed for the water phase. Similarly, when the channel scale is reduced to 21 nm, the final deviation degree is 54.1% for oil, while it exceeds 80% for water. Furthermore, when there is a change in the fluid type, such as its composition or properties, the intensity of the interaction between the fluid and the solid surface changes accordingly. This change in interaction has a significant impact on the flow behavior and cannot be ignored in a confined space.

### 2.2. Flow Resistance of the Different Medium

The deviation degree of water at the same scale is obviously greater than that of the oil. The normalized resistance coefficient is used to further compare the flow resistance characteristics of two fluids.

**Figure 3:**
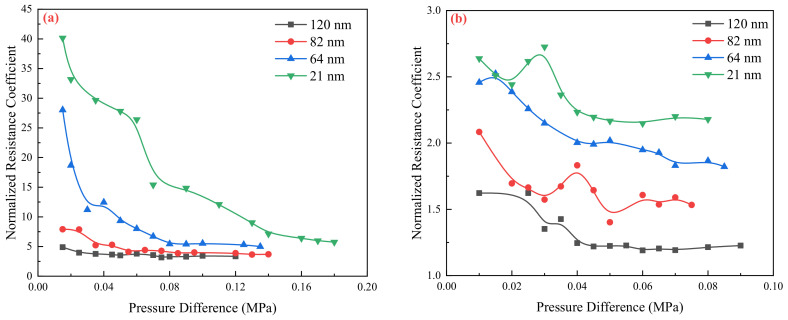
Normalized flow Drag coefficient under different pressures: (**a**) Deionized water; (**b**) Silicon oil.

Theoretically, the normalized drag coefficient should be one if the confinement effect is ignored. However, as shown in Figure 3, the flow resistance coefficient of water and oil is greater than one. Additionally, it mentions that the flow resistance coefficient of the water phase in the nanochannel is larger than that of the oil phase. The range of flow resistance coefficients mentioned (3.7–40.2 for water and 1.2–2.4 for oil) indicates that the extent of the confinement effect varies depending on the channel size and the fluid types. Smaller channel sizes lead to higher flow resistance coefficients, suggesting stronger confinement effects.

In this study, the alumina nanochannels have strong hydrophilicity, meaning that water molecules have a stronger interaction with the membrane’s surface compared to oil molecules. Consequently, the impact of the solid wall on water flow is greater than that of oil flow at the same scale. Based on these phenomena, it can be concluded that when investigating fluid motion in confined spaces, the influence of wettability on flow cannot be disregarded. The interaction strength between fluid and solid walls plays a significant role in determining the flow characteristics.

## 3. Discussions

### 3.1. Heterogeneous Viscosity Flow Model

**Figure 4:**
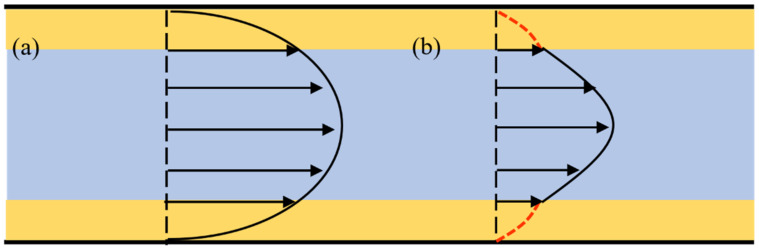
Velocity profile: (**a**) Hagen–Poiseuille equation; (**b**) Heterogeneous viscosity flow model.

According to traditional fluid mechanics theory, the laminar velocity distribution in a long straight pipe is described as a rotating paraboloid around the pipe axis, as depicted in Figure 4a. However, molecular simulations have revealed that in confined spaces, fluid molecules tend to arrange themselves in ordered stacking structures near the wall. This phenomenon creates variations in density and viscosity between the fluid close to the wall and the bulk phase, leading to unique flow behaviors in nanochannels.

Therefore, to effectively describe the flow characteristics of the fluid in nanochannels, a heterogeneous viscosity flow model is proposed. It divides the channel into two parts: the bulk phase region and the boundary region. This division is shown in Figure 4b, where the yellow part represents the boundary area with a thickness of h. Since the fluid viscosities in the two regions are different, there are two velocity profiles.

The velocity distribution in the boundary region is:

(1) $$u_{BC}=\frac{R^{2}-r^{2}}{4\mu_{BC}}\frac{\Delta P}{L}+C_{1}$$

The velocity distribution in the bulk region is:

(2) $$u_{bulk}=\frac{R^{2}-r^{2}}{4\mu_{bulk}}\frac{\Delta P}{L}+C_{2}$$

where $u_{BC}$ and $u_{bulk}$ represent the velocity of the boundary region and the bulk region, respectively; $\mu_{BC}$ and $\mu_{bulk}$ represent the fluid viscosity of the boundary region and the bulk region, respectively; C_1_ and C_2_ are parameters related to the fluid properties in the boundary region. To determine C_1_ and C_2_, boundary conditions need to be added. When *r* = R, $u_{BC}$ = 0; when *r* = R − h, $u_{BC}$ = $u_{bulk}$. Substitute the boundary conditions into Equations (1) and (2):

$$C_{1}=0$$

$$C_{2}=\left(2Rh-h^{2}\right)\left(\frac{1}{\mu_{BC}}-\frac{1}{\mu_{bulk}}\right)\frac{\Delta P}{L}$$

In summary, the velocity distribution of the boundary region is:

(3) $$u_{BC}=\frac{R^{2}-r^{2}}{4\mu_{BC}}\frac{\Delta P}{L}$$

The velocity distribution of the bulk region is:

(4) $$u_{bulk}=\frac{R^{2}-r^{2}}{4\mu_{bulk}}\frac{\Delta P}{L}+\left(2Rh-h^{2}\right)\left(\frac{1}{\mu_{BC}}-\frac{1}{\mu_{bulk}}\right)\frac{\Delta P}{L}$$

By integrating the velocity over the entire effective cross-section, the total flow rate in the pipe can be obtained as:

(5) $$Q=\int_{0}^{R-h}u_{bulk}2\pi rdr+\int_{R-h}^{R}u_{BC}2\pi rdr$$

Bring Equations (3) and (4) into Equation (5):

(6) $$Q=2\pi \times \frac{\Delta P}{4L}\times \frac{{\left(h-R\right)}^{2}\left[\mu_{BC}\left(h^{2}-2hR+R^{2}\right)\right]-2\mu_{bulk}\left(h^{2}-2Rh\right)}{4\times \mu_{bulk}\times \mu_{BC}}\;+2\pi \times \frac{\Delta P}{4L}\times \left(\frac{h^{4}}{4\mu_{BC}}-\frac{h^{3}R}{\mu_{BC}}+\frac{h^{2}R^{2}}{\mu_{BC}}\right)$$

Equation (6) takes into account the viscosity and thickness of the boundary region fluid, enabling a more accurate description of fluid flow characteristics within the nanochannel.

### 3.2. Fluid Viscosity and Thickness of the Boundary Region

**Figure 5:**
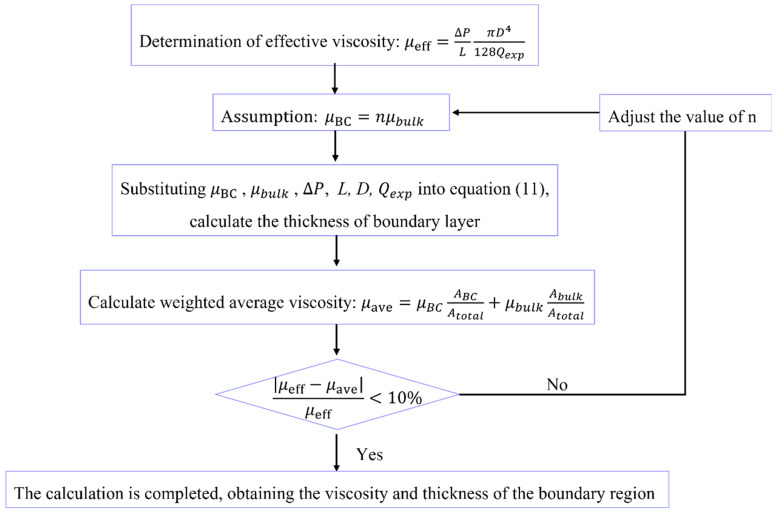
Calculation flowchart of fluid viscosity and thickness of boundary region.

Based on Equation (6) and the measured experimental data, the fluid viscosity and thickness variation characteristics of the boundary region in channels of different sizes can be obtained by combining hypothesis and verification. The specific calculation steps are shown in Figure 5.

(1) Assuming the viscosity of the fluid is a necessary step in calculating the thickness of the boundary region when the fluid viscosity and thickness are unknown. To determine the appropriateness of the assumed fluid viscosity, a standard is required. In this case, the effective viscosity of the fluid can be calculated based on the HP equation and experimentally measured flow rate at different pore sizes and pressures, which serves as a benchmark for subsequent judgment.
(2) Based on the given information, both the water phase and the oil phase are in a flow inhibition state in the nanochannel. This indicates that the viscosity of the boundary layer is greater than the bulk viscosity. Meanwhile, the fluid viscosity is also greater than the effective viscosity determined in the first step. Taking these factors into account, the ratio n of viscosity of boundary fluid and bulk fluid can be estimated.
(3) Input the following parameters into Equation (12), boundary region fluid viscosity, bulk fluid viscosity, pressure gradient, experimental flow rate, and calculate the thickness h of the boundary layer.
(4) After obtaining h, it is crucial to combine the viscosity of the boundary region fluid and bulk fluid to form an average viscosity for comparison with the effective viscosity. Calculate the weighted average viscosity of the fluid in the nanochannel according to Equation (7):

(7) $$\mu_{ave}=\mu_{BC}\frac{A_{BC}}{A_{total}}+\mu_{bulk}\frac{A_{bulk}}{A_{total}}$$

where $\mu_{ave}$ is weighted average viscosity; $A_{BC}$ and $A_{bulk}$ are the cross-sectional areas of the boundary and bulk regions; $A_{total}$ is the cross-sectional area of the entire channel.

(5) Calculate the relative error between the average viscosity and the effective viscosity. If the relative error is less than 10%, it indicates that the assumed boundary fluid viscosity is appropriate, and the calculation can be concluded. However, if the relative error is greater than 10%, it suggests that there is an issue with the initially selected boundary fluid viscosity. In this case, you should go back to step (2) and adjust the value of n to restart the calculation. Continue iterating and adjusting the n value until the relative error is less than 10%.

**Figure 6:**
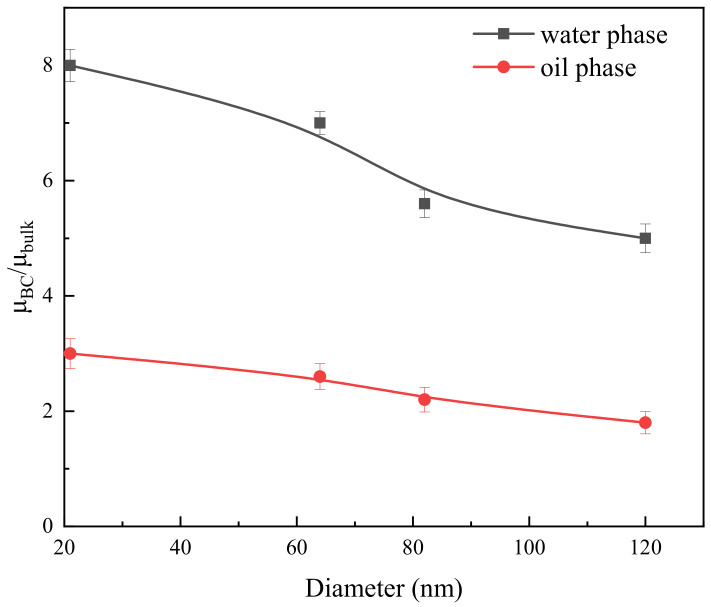
Ratio of boundary fluid viscosity to bulk fluid viscosity in different size channels.

Figure 6 displays the ratio of boundary fluid viscosity to bulk fluid viscosity for both the water phase and oil phase in channels of varying sizes. The variation trend of fluid viscosity in the boundary region of the two fluids is similar. As the channel size increases, the fluid viscosity in the boundary layer gradually decreases. However, there is a more significant difference in viscosity between the boundary and the bulk region fluid for water, compared to the oil phase. Specifically, when the pore size changes within the range of 21 to 120 nm, the n value of the aqueous phase varies from 8 to 5. On the other hand, the n value of the oil phase only ranges from 3 to 1.8. As the channel size increases, the difference between the two gradually decreases. Din et al. investigated the boundary layer viscosity in nanoscale channels using molecular simulation methods [36]. They set the interaction intensity between the fluid and solid wall with a difference of 3.5 times. They observed that as the relative interaction intensity increased, the boundary layer viscosity also increased. This finding is in line with the trend observed in experimental analysis results, suggesting that the calculated boundary layer viscosity using this method is reasonable.

Both channel size and wall wettability are critical factors influencing the viscosity of boundary fluids. The channel walls are highly hydrophilic, meaning they have a strong attraction to water molecules compared to oil molecules. This difference in attraction causes a more substantial viscosity change in the water phase compared to the oil phase within the boundary region. As the spatial scale decreases, the movement of particles within the boundary region becomes more restricted, which leads to an increase in viscosity. In summary, in smaller channels, where the surface-to-volume ratio is larger, even small changes in wettability can have a more significant impact on the effective viscosity of the fluid. It is important to consider this effect when studying fluid flow and designing microfluidic systems or nanofluidic devices where channel dimensions are very small.

On the basis of the fluid viscosity of the boundary region, we can further analyze the variation features of the thickness of the boundary region under different pore sizes and pressures.

**Figure 7:**
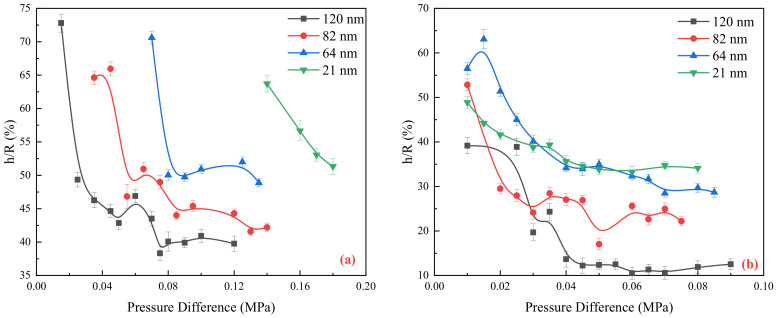
Boundary region thickness variation in different scale channels: (**a**) Deionized water; (**b**) Silicon oil.

Figure 7 illustrates the variation characteristics of the boundary region thickness of the water and oil under different pressures. As the pressure increases, the thickness gradually decreases until it reaches a stable value. Considering the same scale, when the boundary region thickness becomes stable, the thickness of the water phase boundary region is approximately 3.3–1.5 times larger than that of the oil phase boundary region. The boundary region can be divided into two parts. The first part of the boundary region is formed by the relative interaction between the fluid and the solid wall, which is not affected by the pressure gradient and is determined by the characteristics of the fluid (such as type and viscosity) and the properties of the solid wall (such as size, wettability, and roughness). The second part of the boundary region is influenced by the pressure gradient. As the driving force applied to the fluid increases, it can overcome the viscous forces between molecules. The pressure gradient continues to increase, and the thickness of this part of the boundary region decreases until it approaches zero.

### 3.3. Model Application

**Figure 8:**
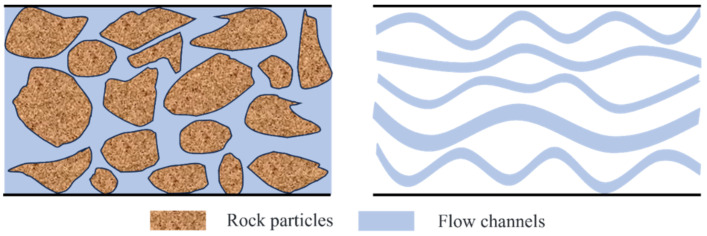
Schematic diagram of capillary bundle model in tight reservoirs.

Microscale effects play a crucial role in the fluid flow behavior of unconventional reservoirs, which are characterized by numerous nanoscale pores and throats. These microstructures result in fluid migration patterns that differ significantly from those observed in macroscopic bulk fluids. Consequently, traditional permeability evaluation models, which overlook microscale effects, fail to accurately capture the effective permeability of such reservoirs. The application of the heterogeneous viscosity model in permeability evaluation, instead of the HP equation, has the potential to enhance the accuracy of model predictions. Taking tight sandstone as an example, its seepage space can be simplified as a series of curved capillaries with different sizes, as shown in Figure 8. Furthermore, it has good fractal characteristics, and the distribution of capillary numbers follows the fractal power-law features. Permeability prediction of tight sandstone can be achieved by combining the capillary bundle model, fractal theory, and heterogeneous viscosity model.

According to the fractal theory, the cumulative total number *N* of pores in a porous medium with pore size greater than or equal to *R* meets the following relationship: [37,38,39,40]

(8) $$N={\left(\frac{R_{max}}{R}\right)}^{D_{f}}$$

(9) $$-dN=D_{f}R_{max}^{D_{f}}R^{-\left(D_{f}+1\right)}dR$$

where *R* is the pore radius, $D_{f}$ is pore fractal dimension.

The total flow rate through the capillary bundle is:

(10) $$Q=\int_{r_{min}}^{r_{max}}q\left(R\right)\cdot dN\left(R\right)$$

Bring Equation (6) and (9) into Equation (10):

(11) $$Q=D_{f}R_{max}^{D_{f}}\cdot \left(2\pi \cdot \frac{\Delta P}{4L_{t}}\right)\int_{r_{min}}^{r_{max}}q\left(R,h,\mu \right)\cdot R^{-\left(D+1\right)}dR$$

Combining Darcy’s Formula:

(12) $$Q=\frac{k}{\mu }A\frac{\Delta P}{L_{0}}$$

The cross-sectional area of the porous medium can be expressed as:

(13) $$A=\frac{A_{p}}{\varphi }=\frac{1}{\varphi }\int_{r_{min}}^{r_{max}}\frac{\pi R^{2}}{4}\cdot dN\left(R\right)=\frac{\pi }{4\varphi }\frac{D_{f}R_{max}^{D_{f}}}{2-D_{f}}\left(R_{max}^{2-D_{f}}-R_{min}^{2-D_{f}}\right)$$

The length of a curved capillary can be expressed as:

(14) $$L_{t}\left(R\right)={\left(2R\right)}^{1-D_{T}}{L_{0}}^{DT}$$

where $D_{T}$ is tortuosity fractal dimension; $L_{0}$ is channel characteristic length.

From the above, it can be concluded that:

(15) $$K=\frac{\pi \mu \cdot D_{f}R_{max}^{D_{f}}\cdot L^{1-D_{T}}}{A\cdot 2^{1-D_{T}}}\cdot \int_{r_{min}}^{r_{max}}q\left(r,h,\mu \right)\cdot R^{-\left(D_{T}-D_{f}-2\right)}dR$$

Table 1 lists the basic physical parameters, pore fractal dimension, and tortuosity fractal dimension of the selected cores. Porosity was obtained using a helium porosity meter, permeability was obtained using a gas permeability meter, and pore fractal dimension and tortuosity fractal dimension were obtained from the rock capillary force curve obtained by high-pressure mercury injection. The pore fractal dimension quantifies the irregularity or complexity of the pore structure. The tortuosity fractal dimension characterizes the degree of tortuosity or winding path of fluid flow through the porous medium. As the permeability decreases, both the pore and tortuosity fractal dimensions exhibit an increasing trend, which points out that the heterogeneity of the core becomes stronger and the distribution of pore and throats becomes more complex.

**Figure 9:**
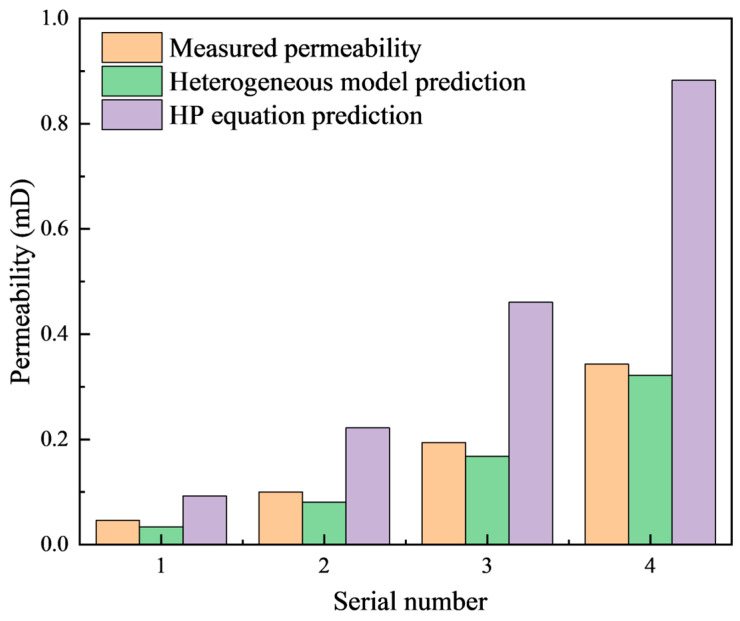
Comparison of the fitting and measured permeability for tight sandstone.

In the prediction model of unconventional reservoir permeability, two key issues need to be noted: the significant confinement effect and complex pore and throat structure. Figure 9 illustrates the disparity between the core permeability predicted by the HP equation and the heterogeneous viscosity model, in comparison to the experimentally measured permeability. The results indicate that there is a significant difference between the permeability calculated using the HP equation and the measured permeability, despite having the same fractal dimension. The heterogeneous viscosity model appears to provide a better representation of the influence of confined space on flow. It shows a good correlation with the measured permeability, especially in cases where the reservoir permeability is low.

## 4. Experimental Section

### 4.1. Materials

**Figure 10:**
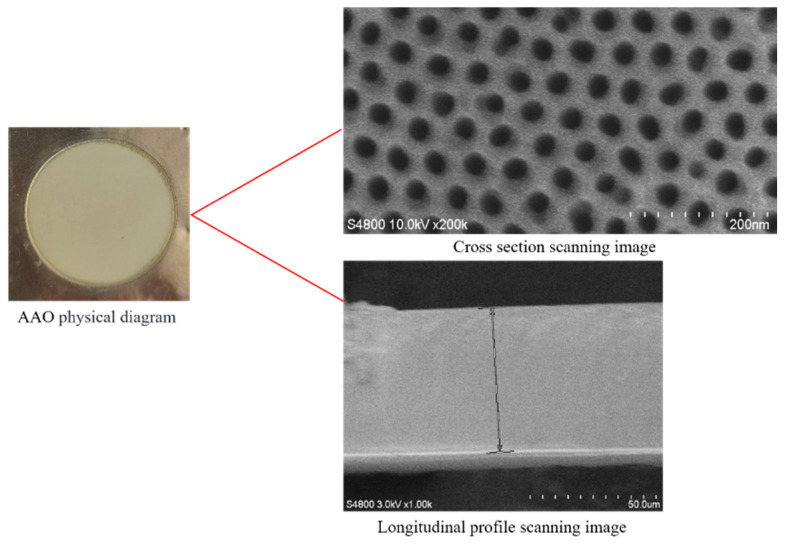
SEM scanning image of AAO.

Due to the extremely low flow rate in a single nanochannel, it is challenging to measure accurately. In this study, an array of nanochannels is used as an experimental platform, which is fabricated by anodizing high-purity aluminum sheets in an acidic solution [11]. The anodized aluminum oxide film is a circular sheet with a diameter of 25 mm and is densely covered with nanoscale pores that are not visible to the naked eye. Scanning electron microscopy is employed to characterize the aluminum oxide film, determining its thickness and pore size for the experiments. Figure 10 shows the SEM scan image of the alumina film used in the experiment. Table 2 lists the diameter and length of nanochannels. The chosen flow medium consists of deionized water and silicone oil. The viscosity of water is 1 mPa·s, and the viscosity of oil is 5 mPa·s.

### 4.2. Experimental Method

#### 4.2.1. Experimental Procedure

**Figure 11:**
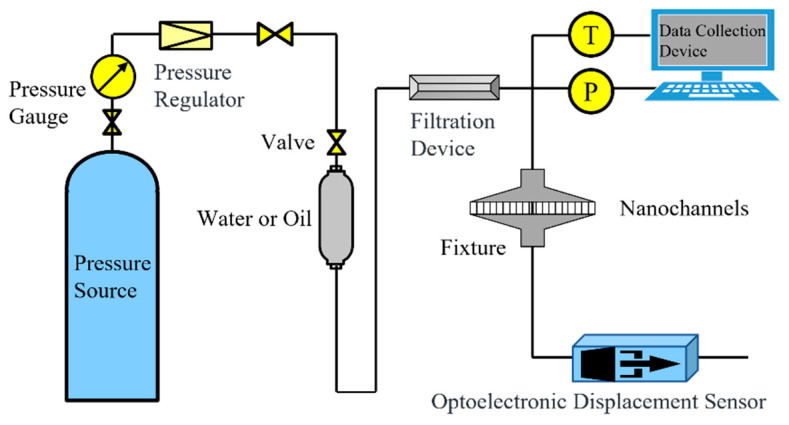
Schematic diagram of the physical simulation apparatus.

The schematic diagram of the experimental setup is illustrated in Figure 11. In this configuration, a steady stream of nitrogen gas is employed as the pressure source. The inlet pressure of the nanochannel is finely adjusted to the desired level by means of a pressure-reducing valve and a pressure-regulating valve. To safeguard against membrane rupture, a purpose-built circular fixture is meticulously designed to secure the membrane from both ends, thereby ensuring even distribution of pressure. The experimental procedure unfolds as follows:

(1) Before commencing the experiment, it is imperative to verify that the intermediate container remains clean and free from any contaminants. Furthermore, a sealing test must be conducted to ascertain the absence of any leaks at the various device connections. (2) Gently and gradually regulate the system pressure to facilitate the gradual entry of the liquid into the nanochannel. Once a stable flow is achieved, record the pressure at the inlet end using the pressure acquisition device. Then, connect a measuring capillary tube with a diameter of 1 mm at the outlet of the nanochannel. Inject a bubble into a fixed position in the measuring tube, and the flow rate is obtained by measuring the displacement of the gas–liquid interface and its corresponding time. By passing the measuring tube through the photodiode, when it senses the gas–liquid interface, the photodiode is triggered to start automatic timing. When the gas–liquid interface reaches the predetermined displacement, the photodiode triggers a signal to terminate timing. This measurement method not only eliminates human timing errors but also enhances measurement accuracy. (3) Once the flow has reached a stable state, proceed to modify the displacement pressure. Repeat the above procedure and record the experimental data under varying pressures. To minimize errors, it is recommended to conduct multiple measurements at each pressure point. The designated pressure range spans from 0 to 0.17 MPa. (4) Calculate the total flow rate through the nanoarray based on displacement, time, and the cross-sectional area of the capillary connected to the outlet of the nanochannel. By combining the number of nanopores in a single film measured by scanning electron microscopy, it is feasible to compute the flow rate within an individual nanochannel.

(16) $$Q_{exp}=\frac{S}{t}\cdot \frac{A}{N}$$

where $Q_{exp}$ is experimental flow rate, *S* is the displacement of the fluid in the capillary; *t* is the duration of the measurement; *A* is the cross-sectional area of the capillary; and *N* is the total number of nanochannels within the alumina membrane.

#### 4.2.2. Data Analysis Method

If the confinement effect is ignored, the relationship between pressure difference and flow rate in a straight circular tube follows the Hagen–Poiseuille equation:

(17) $$Q_{HP}=\frac{\pi D^{4}}{128\mu }\frac{\Delta P}{L}$$

where $Q_{HP}$ is theoretical Flow, m^3^/s; *D* is pipe diameter, m; *L* is pipe length, m; μ is fluid viscosity, Pa·s; ΔP is the pressure difference between the inlet and outlet ends, Pa. The impact of confined space on flow can be assessed by comparing the differences between experimental flow and theoretical flow. Quantitatively demonstrating these differences can be achieved by calculating the degree of deviation D of the experimental flow.

(18) $$D=\frac{Q_{HP}-Q_{exp}}{Q_{HP}}$$

In addition, to further analyze the reasons for the significant differences in deviation degree among different types of fluids in a nanochannel, the normalized flow drag coefficient C* can be utilized to characterize the flow resistance of various fluids [41]:

(19) $$Re=\frac{\rho uD}{\mu }$$

(20) $$f=\frac{\Delta P}{\frac{{pu}^{2}}{2}\frac{L}{D}}$$

(21) $$C^{*}=\frac{{\left(Ref\right)}_{exp}}{{\left(Ref\right)}_{HP}}=\frac{Q_{HP}}{Q_{exp}}$$

where Re is Reynolds number, ρ. is the fluid density, kg/m^3^; u is average speed, m/s; f is resistance coefficient. It should be pointed out that in the subsequent data analysis, except for the basic data, the spline curves were employed to distinctly illustrate the variation patterns of pertinent parameters across different scales of nanochannels.

## 5. Conclusions

In this study, flow characteristics of water and oil in hydrophilic alumina nanochannels were investigated with diameters ranging from 21 nm to 120 nm, and a heterogeneous viscosity flow model considering fluid viscosity was proposed. Compared to the theoretically predicted flow rate, both water and oil in confined spaces experience notable flow suppression phenomena. As the channel size decreases, the deviation in flow rate becomes more pronounced. The maximum deviation of water is 83.6%. At the same scale, the flow rate deviation of the water phase is considerably greater due to its stronger interaction with the solid walls, which results in higher flow resistance for the water phase.

The flow region can be divided into two parts: the boundary region and the bulk phase region. The fluid viscosity in the boundary region is primarily influenced by the pore size and wall wettability. As the pore size decreases or non-wetting fluid transitions to wetting fluid, the viscosity of the boundary fluid increases, especially in the case of small pore size, which will magnify the influence of wettability. As the pressure increases, the thickness of the boundary region gradually decreases. The stability of the boundary region thickness is primarily determined by the strength of the interaction between the fluid and solid surfaces. The proposed heterogeneous flow model can be effectively applied in the evaluation of fluid flow in unconventional reservoirs, providing important insights into explaining abnormal flow phenomena and determining parameters in related mathematical models.

## Figures and Tables

**Table 1:** Basic parameters of rock samples.

Serial Number	Porosity/%	Permeability/mD	D_f_	D_T_
1	5.14	0.046	1.35	1.52
2	6.32	0.1	1.27	1.48
3	8.45	0.194	1.17	1.44
4	10.19	0.343	1.08	1.42

**Table 2:** Parameters of the anodized aluminum oxide film used in the experiments.

**Pore Diameter/nm**	21	64	82	120
**Pore Length/μm**	56.4	88.1	93.7	113

## Data Availability

The data presented in this study are available in article.
